# Pregnancy outcomes in women with anti-β2-glycoprotein I/human leukocyte antigen-DR autoantibodies receiving assisted reproductive technology: a prospective cohort study

**DOI:** 10.3389/fimmu.2025.1626862

**Published:** 2025-11-24

**Authors:** Yosuke Ono, Shinichiro Wada, Yuta Kobayashi, Maki Ogi, Yoshiyuki Fukushi, Kenji Tanimura, Hisashi Arase, Osamu Yoshino, Hideto Yamada

**Affiliations:** 1 Department of Obstetrics and Gynecology, University of Yamanashi, Yamanashi, Japan; 2 Department of Obstetrics and Gynecology, Teine Keijinkai Hospital, Sapporo, Japan; 3 Department of Obstetrics and Gynecology, Kobe University Graduate School of Medicine, Chuo-ku, Kobe, Japan; 4 World Premier International Immunology Frontier Research Center, Research Institute for Microbial Diseases, Osaka University, Suita, Osaka, Japan; 5 Center for Recurrent Pregnancy Loss, Teine Keijinkai Hospital, Sapporo, Japan

**Keywords:** autoantibody, β2-glycoprotein I, HLA-DR, embryo transfer, implantation failure, low dose aspirin, unfractionated heparin, pregnancy

## Abstract

**Objective:**

This study aimed to assess whether anti-β2-glycoprotein I (β2GPI)/human leukocyte antigen (HLA)-DR autoantibodies are associated with pregnancy outcomes in women with infertility receiving assisted reproductive technology (ART) in relation to antithrombotic therapies.

**Methods:**

In this multicenter prospective cohort study, levels of anti-β2GPI/HLA-DR autoantibodies were measured in 194 women with infertility, who subsequently received embryo transfer (ET). The rates of clinical pregnancy, biochemical pregnancy loss, live birth, and miscarriage were assessed in relation to antibody positivity and antithrombotic treatments. The primary outcome was to evaluate how antithrombotic treatments were associated with pregnancy outcomes in women with anti-β2GPI/HLA-DR antibodies receiving ART. The treatment modality for the antibody-positive group was determined for each ET at the discretion of the attending physician.

**Results:**

Finally, 30 women in the antibody-positive group and 123 in the antibody-negative group were analyzed. The prevalence of recurrent implantation failure in the antibody-positive group (40.0%, 12/30) was higher than that in the antibody-negative group (20.3%, 25/123; p = 0.024). The clinical pregnancy rate per ET tended to be lower in the antibody-positive group than in the antibody-negative group (30.4%, 21/69 vs 43.6%, 92/211; p=0.053), while the implantation rate per embryo was significantly lower in the antibody-positive group than in the antibody-negative group (26.3%, 21/80 vs 39.0%, 92/236; p=0.040). Among women in the antibody-positive group, low-dose aspirin (LDA), LDA plus unfractionated heparin (UFH), and non-LDA/non-UFH treatments were given in 30, 5, and 34 ET cases, respectively. The clinical pregnancy (42.9%, 15/35 ET vs. 17.6%, 6/34 ET; p = 0.044) and live birth (37.1%, 13/35 ET vs. 11.8%, 4/34 ET; p = 0.030) rates were higher in treated-group with LDA/UFH than non-LDA/non-UFH group. LDA/UFH treatment was independently associated with higher clinical pregnancy rates (adjusted odds ratio, 3.34; 95% confidence interval, 1.02–12.3; p = 0.047). Immunofluorescent staining showed coordinated expression of β2GPI and HLA-DR antigens on the epithelial cells of the eutopic endometrium in the antibody-positive group.

**Conclusion:**

LDA/UFH treatment may be associated with higher clinical pregnancy and live-birth rates in women positive for anti-β2GPI/HLA-DR antibodies receiving ART.

## Introduction

Implantation is a crosstalk between the embryo and endometrium and represents the first crucial step in a successful pregnancy. Implantation failure occurs when the embryo fails to attach to or invade the endometrium. However, its pathophysiology remains insufficiently elucidated and is one of the reasons for the stagnation in the success rates of assisted reproductive technology (ART) (1). Implantation failure is considered primarily caused by embryonic abnormalities, specifically aneuploidy (2). Advancements in preimplantation genetic testing for aneuploidy (PGT-A), such as blastocyst culture and embryo vitrification, have led to reductions in the risk of implantation failures associated with chromosomal abnormalities (3–5). Recently, a study indicated that if diploid embryos with a normal chromosome structure can be transferred, the implantation rate remains the same regardless of the maternal age (6). Nevertheless, implantation failure still occurs, suggesting the important role of the uterine environment for embryo implantation. Several factors on the endometrial side, such as immunological factors, thrombophilia, endometrial microbiome, chronic endometritis, endometrial receptivity, and anatomical abnormalities, were investigated as potential contributors to implantation failure (7). However, the target factors, diagnostic methods, and treatment strategies for uterine factor-related implantation failure have yet to be established.

Recently, a new concept has emerged, suggesting that autoantibodies are released in response to misfolded protein complexes that form with human leukocyte antigen (HLA) class II molecules carrying alleles associated with disease susceptibility (8, 9). Among these autoantibodies (called neoself antibodies), Tanimura et al. recognized the involvement of anti-β2GPI/HLA-DR antibody, which targets the complex formed by β2-glycoprotein I (β2GPI) and HLA-DR, in the pathogenesis of recurrent pregnancy loss (RPL) (10) and pregnancy complications associated with antiphospholipid syndrome (APS) (11). They highlighted that β2GPI/HLA-DR antigens are expressed on the endothelial cells within the decidua of miscarried placentas from women with obstetric APS and that autoantibodies targeting these antigens may disrupt placental function.

Some studies have indicated a high prevalence of different autoantibodies in women with infertility (12, 13); however, a causal link to implantation failure has not been determined. Because the mechanism of anti-β2GPI/HLA-DR antibodies, which are produced in response to increased antigen expression triggered by infections or inflammation, differs from that of conventional autoantibodies, investigating the involvement of these antibodies in implantation is considered worthwhile. Recently, we demonstrated that 17.9% of women with infertility and 15.5% of women undergoing ART tested positive for anti-β2GPI/HLA-DR antibodies. Moreover, 28.9% of women with infertility complicated by severe endometriosis and 27.8% with recurrent implantation failure (RIF) tested positive for anti-β2GPI/HLA-DR antibodies, and both conditions showed independent association with positivity to these antibodies (14). In our previous study, we measured β2GPI/HLA-DR antibodies only in women with infertility, particularly those undergoing ART (14), and did not include concurrent measurements in healthy controls; therefore, differences in antibody levels between patients and healthy women cannot be directly inferred. However, considering that the reference cutoff for anti-β2GPI/HLA-DR antibodies is defined as the 99th percentile of the distribution in healthy controls (15), the abnormally high titers in patients may be associated with infertility. A study also implicated this antibody in endometrial thrombus formation and endothelial injury (10). Accordingly, in the present prospective study, serum anti-β2GPI/HLA-DR antibodies were quantified before ART, and pregnancy outcomes were evaluated in relation to antibody positivity and the use of antithrombotic therapy.

## Materials and methods

### Patient enrollment

This prospective observational study was conducted between July 2020 and September 2024 at the Teine Keijinkai Hospital and the University of Yamanashi Hospital in accordance with the principles outlined in the Declaration of Helsinki. It was approved by the institutional review boards of both institutions (Approval nos. 2-020090–01 and 2-023209-00, respectively). The study enrolled women with infertility who were scheduled for ART and underwent serum anti-β2GPI/HLA-DR antibody measurements at either facility between July 2020 and July 2023, and their pregnancy outcomes were monitored until September 2024. Women with missing data and a history of chronic hypertension, diabetes mellitus, endocrine diseases, cardiovascular diseases, or malignancies were excluded.

### Study protocol and clinical management for women undergoing ART

ART indications were determined by conventional tests for infertility, including transvaginal ultrasonography, hysterosalpingography, semen analysis, and hysteroscopy, and history of fertility treatment. The underlying etiologies for infertility were classified into ovulatory, uterine, fallopian tubal, male-related, and unknown factors. Endometriosis was diagnosed by magnetic resonance imaging and/or based on postoperative pathological findings.

Women with ART who underwent measurements of serum anti-β2GPI/HLA-DR antibody levels were divided into women who tested positive for anti-β2GPI/HLA-DR antibodies (antibody-positive group) and women who tested negative (antibody-negative group). These groups were compared based on their backgrounds and underlying etiologies for infertility. The cutoff value for anti-β2GPI/HLA-DR antibody positivity was determined based on the 99th percentile of the distribution in healthy controls (normal, <73.3 U) (15). The following clinical data were collected from the electronic medical records of all participants: maternal age, gravidity and parity, body mass index (BMI), history of RIF, history of RPL, and ART outcomes, including clinical pregnancy rate, biochemical pregnancy rate, live-birth rate per embryo transfer (ET), and miscarriage rate per clinical pregnancy. RIF was defined as three or more implantation failures following *in vitro* fertilization and ET (16).The clinical pregnancy rate was defined as the proportion of embryo transfers (ETs) with a gestational sac (GS). The implantation rate was defined as the proportion of embryos transferred that resulted in a GS. For both implantation and clinical pregnancy rates, GS was confirmed by transvaginal ultrasonography. To increase the sensitivity of ART outcome analyses, implantation and clinical pregnancy rates were also evaluated per single ET (SET). If multiple SETs failed to achieve pregnancy or when advanced maternal age was a concern, up to two embryos were transferred per cycle. During the study period, some patients in both the antibody-positive and antibody-negative groups underwent more than one ET. As a result, 69 ET cycles were performed among 30 antibody-positive patients and 211 ET cycles among 123 antibody-negative patients. These numbers indicate the total number of ET cycles, not the total number of embryos transferred.

In this study, the embryos that could be transferred were considered grade ≥3 at the 6–8 cell stage by the Veek classification and grade 3BB or higher by the Gardner classification for blastocyst transfers.

For the anti-β2GPI/HLA-DR antibody-positive group, each attending physician offers information about treatment options, for example, no treatment, antiplatelet therapy, and unfractionated heparin (UFH) therapy. If antiplatelet therapy was deemed necessary by the attending physician, low-dose aspirin (LDA, 81–100 mg/day) therapy was initiated at least 1 week before ET with the patient’s consent. When UFH was used in conjunction with LDA, a prophylactic dose of UFH (5,000 U twice daily) was started at the day of ET or as soon as pregnancy was confirmed, and was continued until 36 weeks of gestation. Serum levels of anti-β2GPI/HLA-DR antibodies were measured at the Revorf Co., Ltd. (currently AOI Biosciences Co., Ltd., Tokyo, Japan) following previously described standardized methods. All measurements were performed in duplicate, and the mean value was defined as the anti-β2GPI/HLA-DR antibody level of the sample. Serum levels of anti-β2GPI/HLA-DR antibodies ≥73.3 U are considered positive.

### Immunofluorescence analysis

Eutopic endometrial tissue sample was obtained during the secretory phase from a 37-year-old woman who tested positive for anti-β2GPI/HLA-DR antibodies and had a history of RIF. The tissue was embedded in paraffin, sectioned at a thickness of 4 µm, and mounted on glass slides. The sections were deparaffinized in xylene, rehydrated through a graded ethanol series, and rinsed with distilled water. Antigens were retrieved by heating the sections in 10 mM sodium citrate buffer (pH 6.0) using a 600-watt microwave for 15 min, followed by cooling to room temperature for approximately 40–45 min. Immunofluorescence staining was conducted according to the manufacturer’s instructions. Anti-APOH (β-2-glycoprotein I; dilution 1:200; HPA001654, Atlas Antibodies, Stockholm, Sweden) and anti-HLA-DRA (major histocompatibility complex class II DR α; dilution 1:1,000; HPA053176, Atlas Antibodies) were used as primary antibodies. The sections were incubated with the primary antibodies overnight at 4 °C and then with the secondary antibody goat anti-rabbit IgG conjugated with Alexa Fluor 488 (ab150077, Abcam, Tokyo, Japan). Nuclei were counterstained with 4′,6-diamidino-2-phenylindole (1:500). For the negative controls, rabbit IgG was utilized instead of the primary antibody. Fluorescence images were captured using a Keyence BZ-X700 microscope (Keyence Corporation, Osaka, Japan).

### Statistical analysis

Study data were analyzed using JMP version 16 (SAS Institute Inc., Cary, NC, USA). Student’s t-test and Fisher’s exact test were employed when comparing the clinical backgrounds between the anti-β2GPI/HLA-DR antibody-positive and antibody-negative groups. Fisher’s exact test was used to compare underlying etiologies and diseases between the two groups. Normality was assessed, and parametric (Student’s t-test) or nonparametric (Mann–Whitney U-test) methods were applied as appropriate. A multivariable logistic regression analysis was also performed in the antibody-positive cohort (n=30, a total of 69 ETs) to evaluate the independent association of LDA/UFH treatment with clinical pregnancy per ET. Variables entered into the model included age, BMI, and a history of RIF, with LDA/UFH treatment as the primary variable of interest. Significance was defined as *p* < 0.05. For comparisons with p > 0.05 that suggested a trend, 95% CIs were additionally computed and reported in **Supplementary Table 1** (items marked with ^†^).

## Results

### Study population

**Figure 1:**
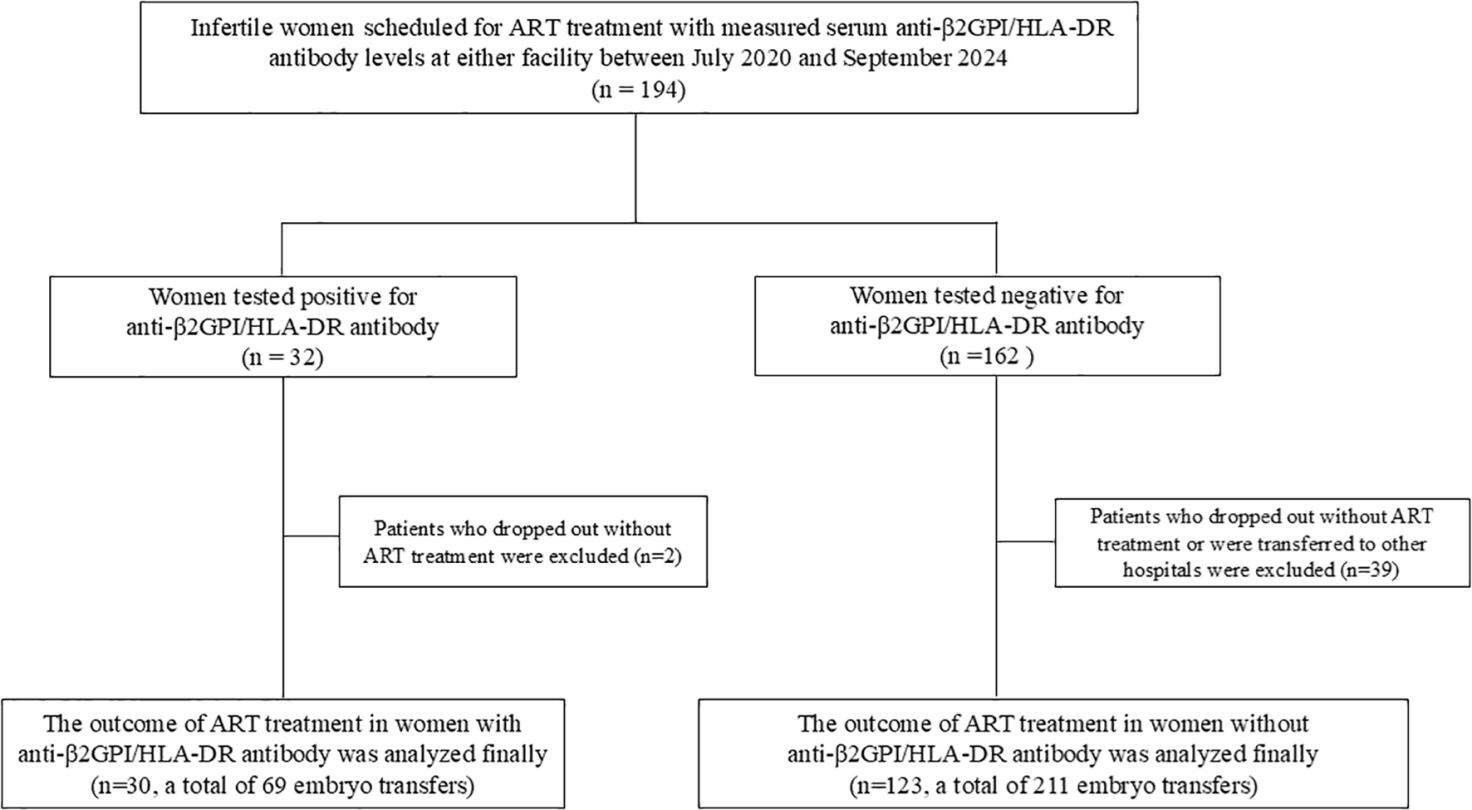
Flow diagram of the participants and their ART outcomes included in the final analysis. ART, assisted reproductive technology.

From July 2020 to July 2023, serum anti-β2GPI/HLA-DR antibody levels (normal, <73.3 U) were measured in 194 women undergoing ART, of whom 32 (16.5%) tested positive for anti-β2GPI/HLA-DR antibodies. However, 2 of the 32 women and 39 of 162 women in the antibody-negative group were excluded because they did not have transferable high-quality embryos that met the transfer criteria, or they transferred to other hospitals. Finally, 30 women who tested positive for anti-β2GPI/HLA-DR antibodies (69 ETs) and 123 who tested negative (211 ETs) were analyzed. Among the ETs, 11 of 69 in the antibody-positive group and 25 of 211 in the antibody-negative group were two-embryo transfers, with the remainder being single-embryo transfers (58 and 186, respectively) (**Figure 1**). Among women in the antibody-positive group, LDA treatment was given in 30 ETs, LDA plus UFH in 5, and non-LDA/non-UFH in 34.

### Clinical characteristics and pregnancy outcomes in the anti-β2GPI/HLA-DR antibody-positive and negative groups

**Table 1** shows the clinical backgrounds of 30 women in the antibody-positive group (antibody value; median, 110.4 U; range, 73.4–162,800 U) and 123 in the antibody-negative group (median, 16.5 U; range, 0–70.1 U). The prevalence of RIF history was higher in the antibody-positive group (40.0%, 12/30) than in the antibody-negative group (20.3%, 25/123; p = 0.024), whereas age, BMI, gravidity, parity, underlying etiologies for infertility and infertility-associated diseases, and prevalence of RPL were not significantly different between the antibody-positive and antibody-negative groups (**Table 1**). If a woman had multiple etiologies/diseases, each was counted separately. The pregnancy outcomes of each ET in the antibody-positive group are shown in **Table 2**.

**Table 1:** Clinical backgrounds of ART women who tested positive or negative for anti-β2GPI/HLA-DR antibody.

Clinical backgrounds	Women with the antibody n = 30	Women without the antibody n = 123	P value
Age, years	38 (31-43)	37 (27-45)	0.611
Body mass index, kg/m2	21.1 (16.8-35.2)	21.5 (16.2-41.1)	0.389
Gravidity	2 (0-4)	1 (0-5)	0.617
Parity	0 (0-2)	0 (0-2)	0.245
Clinical etiologies/diseases associated with infertility			
Tubal factors	10 (33.3%)	36 (29.3%)	0.663
Uterine factors	11 (36.7%)	31 (25.2%)	0.207
Ovulatory factor	5 (16.7%)	27 (22.0%)	0.523
Unexplained factor	2 (6.7%)	19 (15.4%)	0.338
Male factor	5 (16.7%)	22 (17.9%)	0.912
Endometriosis	10 (33.3%)	28 (22.8%)	0.23
History of recurrent implantation failure	12 (40.0%)	25 (20.3%)	0.024
History of recurrent pregnancy loss	8 (26.7%)	14 (11.4%)	0.064
anti-β2GPI/HLA-DR antibody value, U	110.4 (73.4–162800)	16.5 (0-70.1)	< 0.001

Data are expressed as median (range) or number (percentage). ART, assisted reproductive technology. Statistical significance was set at *P* < 0.05.

**Table 2:** Treatment modalities and pregnancy outcomes per each embryo transfer in women tested positive for anti-β2GPI/HLA-DR antibody.

Case	Treatment	anti-β2GPI /HLA-DR levels (U)	Age (y)	BMI (kg/m2)	Gravidity (no.)	Parity (no.)	RPL	Endometriosis	Clinical pregnancy	Pregnancy outcome	Pregnancy complication
1	LDA	162800.0	40	20.6	2	2	–	–	–	–	–
2	LDA	162800.0	40	20.6	2	2	–	–	–	–	–
3	LDA	162800.0	41	20.6	2	2	–	–	–	–	–
4	LDA	1285.9	43	25.9	4	2	+	–	–	–	–
5	LDA	1285.9	43	25.9	4	2	+	–	–	–	–
6	LDA	117.2	35	20.7	0	0	–	+	+	SA (6w)	–
7	LDA	117.2	35	20.7	0	0	–	+	–	–	–
8	LDA	107.4	28	16.8	1	1	–	–	+	Live birth (38w)	–
9	LDA	102.8	31	32	2	1	–	+	+	Live birth (38w)	PPH
10	LDA	102.8	32	32	3	2	–	+	+	PD (36w)	HDP (33w)
11	LDA	81.8	42	22.7	2	0	+	–	+	Live birth (37w)	–
12	LDA	146.8	35	30	2	0	+	–	–	–	–
13	LDA	146.8	36	30	2	0	+	–	+	Live birth (35w)	PD (35w)
14	LDA	84.6	30	21.9	1	0	–	+	+	–	–
15	LDA	407.5	37	19.7	1	1	–	+	–	–	–
16	LDA	862.9	39	21.5	1	0	–	–	+	Live birth (40w)	–
17	LDA	139.8	39	20.3	2	0	+	–	–	–	–
18	LDA	139.8	39	20.3	2	0	+	–	+	SA (8w)	–
19	LDA	139.8	40	20.3	3	0	+	–	–	–	–
20	LDA	79.5	40	26.7	1	0	–	–	–	–	–
21	LDA	78.7	33	18	0	0	–	+	–	–	–
22	LDA	281.3	33	18.7	6	1	–	–	+	Live birth (41w)	–
23	LDA	113.3	41	20.1	1	0	+	–	–		–
24	LDA	113.3	42	20.1	1	0	+	–	–		–
25	LDA	113.3	42	20.1	1	0	+	–	+	Live birth (40w)	–
26	LDA	94	35	18.3	1	0	–	+	–		–
27	LDA	94	36	18.3	1	0	–	+	+	Live birth (37w)	–
28	LDA	77.6	42	28.4	0	0	–	–	–		–
29	LDA	314	41	20.3	0	0	–	–	–		–
30	LDA+UFH	117.2	35	20.7	0	0	–	+	+	Live birth (38w)	Placenta previa, GDM, PPH
31	LDA+UFH	79.5	40	26.7	2	1	–	–	+	Live birth (26w)	DD twin(24w) IUFD (1 fetus), placental abruption (26w)
32	LDA+UFH	79.5	41	26.7	2	1	–	–	–	–	–
33	LDA+UFH	79.5	41	26.7	2	1	–	–	–	–	–
34	LDA+UFH	79.5	41	26.7	2	1	–	–	+	Live birth (38w)	–
35	None	162800.0	40	20.6	2	2	–	–	–	–	–
36	None	1285.9	43	25.9	4	2	+	–	–	–	–
37	None	117.2	35	20.7	0	0	–	+	–	–	–
38	None	117.2	35	20.7	0	0	–	+	–	–	–
39	None	146.8	35	30	2	0	+	–	–	–	–
40	None	146.8	35	30	2	0	+	–	–	–	–
41	None	150.7	3	22.7	2	1	+	+	+	+	PPH
42	None	407.5	37	19.7	1	1	–	+	–	Live birth (39w)	–
43	None	407.5	37	19.7	1	1	–	+	–	–	–
44	None	218.5	37	20	0	0	–	–	–	–	
45	None	218.5	37	20	0	0	–	–	–	–	
46	None	73.4	32	19.5	1	0	–	–	–	–	–
47	None	73.4	32	19.5	1	0	–	–	+	Live birth (33w)	Preeclampsia, HELLP syndrome (33w)
48	None	76.4	39	31.4	2	1	–	–	+	Live birth (38w)	–
49	None	277.2	28	35.2	0	0	–	–	–	–	–
50	None	277.2	28	35.2	0	0	–	–	–	–	–
51	None	139.8	40	20.3	3	0	+	–	–	–	–
52	None	79.5	40	26.7	1	0	–	–	–	–	–
53	None	100.2	32	19.1	0	0	–	+	–	–	–
54	None	100.2	32	19.1	0	0	–	+	+	Live birth (38w)	–
55	None	92.7	39	20.3	1	0	–	–	–	–	–
56	None	92.7	39	20.3	1	0	–	–	–	–	–
57	None	93.3	38	18.6	1	1	–	+	–	–	–
58	None	93.3	38	18.6	1	1	–	+	–	–	–
59	None	147.7	41	30.5	3	0	+	+	–	–	–
60	None	281.3	33	18.7	4	1	+	–	+	SA (8w)	–
61	None	281.3	33	18.7	5	1	+	–	+	SA (6w)	–
62	None	113.3	41	20.1	1	0	–	–	–	–	–
63	None	113.3	41	20.1	1	0	–	–	–	–	–
64	None	94	35	18.3	1	0	–	+	–	–	–
65	None	94	35	18.3	1	0	–	+	–	–	–
66	None	77.6	42	28.4	0	0	–	–	–	–	–
67	None	314	41	20.3	0	0	–	–	–	–	–
68	None	126.3	40	24.1	1	0	–	–	–	–	–
69	None	126.3	40	24.1	1	0	–	–	–	–	–

RPL, recurrent pregnancy loss; SA, spontaneous abortion; PPH, postpartum heamorrhage; IUFD, intra uterine fetal death; GDM, gestational diabetes mellitus; PD, preterm deliverly; HDP, hypertension disorder of pregnancy; W, week.

Among 153 women, the clinical pregnancy rate per ET tended to be lower in the antibody-positive group than in the antibody-negative group (30.4%, 21/69 vs 43.6%, 92/211; p = 0.053). The implantation rate per embryo was significantly lower in the antibody-positive group than in the antibody-negative group (26.3%, 21/80 vs 39.0%, 92/236; p = 0.040). However, when restricted to SET, this difference did not reach statistical significance but showed a tendency toward being lower in the antibody-positive group (31.0%, 18/58 vs 44.6%, 83/186; p = 0.067). No differences were found in biochemical pregnancy loss, live birth, or miscarriage rates between groups (**Table 3**). Furthermore, whether serum anti-β2GPI/HLA-DR antibody levels were associated with clinical pregnancy per ET was also examined. Univariable logistic regression treating the antibody titer as a continuous variable did not show a significant association (odds ratio per 1U increase, 1.001; 95% CI, 0.999–1.003; *p* = 0.311; **Supplementary Table 2a**). Consistently, antibody titers did not differ between transfers that did and did not result in clinical pregnancy (median [range], 22.9 U [0–281.3] vs. 23.7 U [0–162,800]; *p* = 0.638; **Supplementary Table 2b**).

**Table 3:** Pregnancy outcomes in 153 ART women with and without anti-β2GPI/HLA-DR antibody.

Pregnancy outcomes	Women with the antibody (n=30, a total of 69 embryo transfers)	Women without the antibody (n=123, a total of 211 embryo transfers)	P value
Clinical pregnancy rate, per embryo transfer	30.4% (21/69)	43.6% (92/211)	0.053
Implantation rate, per single embryo transfer	31.0% (18/58)	44.6% (83/186)	0.067
Implantation rate, per embryo	26.3% (21/80)	39.0% (92/236)	0.04
Biochemical pregnancy rate, per embryo transfer	13.4% (9/69)	8.1% (17/211)	0.317
Live birth rate, per embryo transfer	24.6% (17/69)	34.1% (72/211)	0.214
Miscarriage rate, per pregnancy	19.0% (4/21)	21.7% (20/92)	0.981

In single embryo transfer cycles, implantation rate equals the clinical pregnancy rate per transfer; therefore, only implantation rate per single embryo transfer is shown to avoid redundancy. Data are expressed as percentage (number). ART, assisted reproductive technology.

Among the 30 women in the antibody-positive group, ART outcomes were compared between women with and without LDA/UFH treatment. The clinical pregnancy rate per ET was significantly higher in the treated group than in the untreated group (42.9%, 15/35 vs. 17.6%, 6/34; p = 0.044). The implantation rate per ET did not reach statistical significance but showed a tendency toward being higher in the treated group (34.9%, 15/43 vs. 16.2%, 6/37; p = 0.102). When the analysis was restricted to SET cycles, a similar trend was observed (44.4%, 12/27 vs. 19.4%, 6/31; p = 0.076), although the difference was not significant. The live-birth rate was also significantly higher in the treated group (37.1%, 13/35 vs. 11.8%, 4/34; p = 0.030). The biochemical pregnancy rate per ET also showed a trend toward being higher in the treated than in the untreated group (11.4%, 4/35 vs. 5.9%, 2/34; p = 0.092), although this trend disappeared in the SET-only analysis (14.8%, 4/27 vs. 6.5%, 2/31; p = 0.402). No significant difference was noted in the miscarriage rate between the two groups (13.3% vs. 33.3%; p = 0.544) (**Table 4**). Among the 30 women (a total of 69 ETs) in the antibody-positive group, multivariable logistic regression adjusting for age, BMI, and history of RIF revealed that LDA/UFH treatment was independently associated with higher odds of achieving clinical pregnancy per ET (adjusted odds ratio, 3.34; 95% CI 1.02–12.3; p = 0.047, **Table 5**).

**Table 4:** Pregnancy outcomes in 30 women with the anti-β2GPI/HLA-DR antibody in relation to treatment.

Pregnancy outcomes	LDA/UFH treatment (n=21, a total of 35 embryo transfers)	Non-LDA/Non-UFH treatment (n=22, a total of 34 embryo transfers)	P value
Clinical pregnancy rate, per embryo transfer	42.9% (15/35)	17.6% (6/34)	0.044
Implantation rate, per single embryo transfer	44.4% (12/27)	19.4% (6/31)	0.076
Implantation rate, per embryo	34.9% (15/43)	16.2% (6/37)	0.102
Biochemical pregnancy, per embryo transfer	11.4% (4/35)	5.9% (2/34)	0.092
Biochemical pregnancy rate, per single embryo transfer	14.8% (4/27)	6.5% (2/31)	0.402
Live-birth rate, per embryo transfer	37.1% (13/35)	11.8% (4/34)	0.03
Miscarriage rate, per pregnancy	13.3% (2/15)	33.3% (2/6)	0.544

In single embryo transfer cycles, implantation rate equals the clinical pregnancy rate per transfer; therefore, only implantation rate per single embryo transfer is shown to avoid redundancy. Treatment decisions were made at the level of each embryo transfer rather than per patient; consequently, the same patient could have both treated and untreated cycles. Data are expressed as percentage (number). ART, assisted reproductive technology. LDA, low dose aspirin. UFH, unfractionated heparin.

**Table 5:** Logistic regression analyses for clinical factors and diseases associated with clinical pregnancy.

Variables	Odds ratio (95% Confidence interval)	P value
Age (per 5 years)	0.80 (0.130-0.76)	0.009
Body mass index (per 5 kg/m^2^)	0.97 (0.41-1.84)	0.721
History of repeated implantation failure	0.35 (0.09-1.20)	0.094
LDA/UFH	3.34 (1.02-12.3)	0.047

Odds ratios for continuous variables are expressed per 5-year increase in age and per 5 kg/m² increase in body mass index. LDA, low dose aspirin. UFH, unfractionated heparin.

Of the 37 women with RIF, clinical pregnancy was not significantly different between the antibody-positive and antibody-negative groups (15.8%, 6/38 vs. 18.2%, 8/48; p=0.913). The implantation rate showed no significant difference in either the analysis restricted to SET cycles (13.3%, 4/30 vs. 15.4%, 6/39; p=0.838) or in the per embryo analysis (13.0%, 6/46 vs. 14.0%, 8/57; p=0.914). The biochemical pregnancy rate per ET, live birth rate, and miscarriage rate did not differ significantly between the antibody-positive and antibody-negative groups: biochemical pregnancy (2.6%, 1/38 vs. 16.7%, 8/48; p = 0.072), live birth (13.2%, 5/38 vs. 10.4%, 5/48; p=0.956), or miscarriage (16.7%, 1/6 vs. 37.5%, 3/7; p=0.559) (**Supplementary Table 3**). Among the 12 patients with RIF who were positive for anti-β2GPI/HLA-DR antibodies, the clinical pregnancy rate per ET showed a non-significant trend toward being higher in the LDA/UFH group (29.4%, 5/17 vs. 4.8%, 1/21, p = 0.071). Implantation rates were not significantly different between the LDA/UFH-treated group and the untreated group (per embryo: 21.7%, 5/23 vs. 4.3%, 1/23; p=0.187) No biochemical pregnancies occurred in either group. Live birth (23.5%, 4/17 vs. 6.3%, 1/21) and miscarriage (20.0%, 1/5 vs. 0%, 0/1) rates were not significantly different between groups (**Supplementary Table 4**).

**Figure 2:**
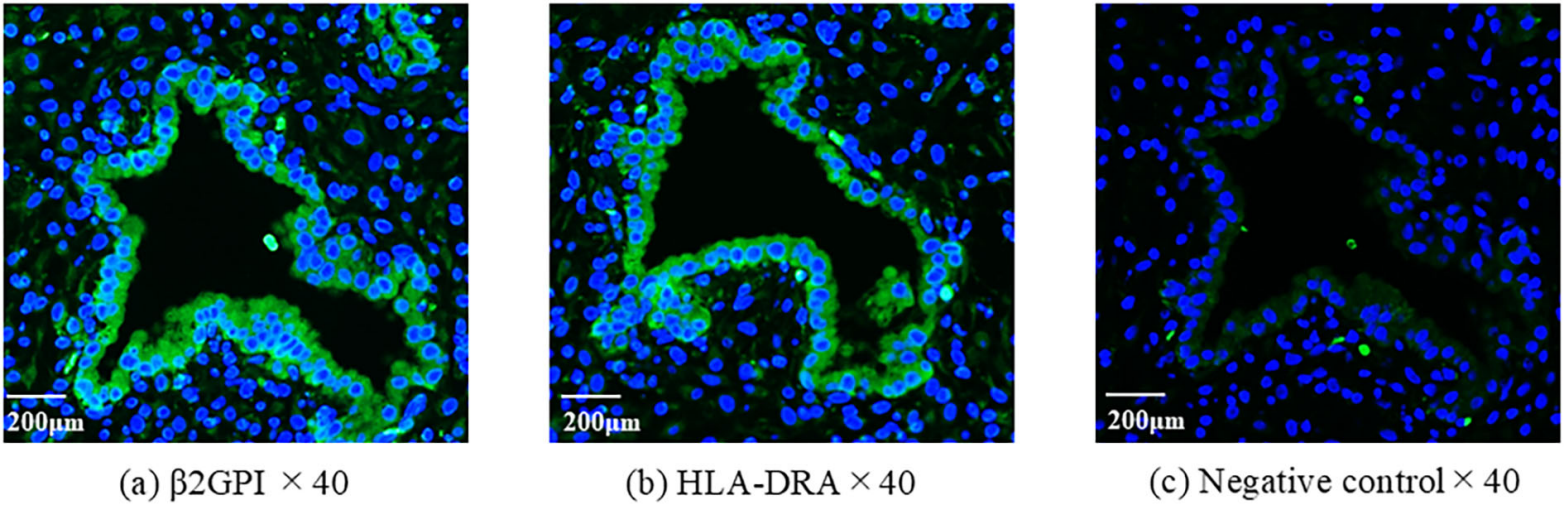
The expression of β2GPI and HLA-DR antigens in eutopic endometrium. Using serial sections of endometrium, antibodies of anti-β2GPI **(a)** or HLA-DR **(b)** were used for immunofluorescent staining. As a second antibody, the mouse anti-rabbit antibody was used. 4′,6-diamidino- 2-phenylindole (DAPI) was used to detect nuclei. The rabbit IgG was used instead of the primary antibody for negative control **(c)**.

Immunofluorescent staining was performed to examine the expression of β2GPI/HLA-DR antigens in the endometrium. β2GPI and HLA-DR were expressed coordinately on the epithelial cells within the eutopic endometrium of a patient with anti-β2GPI/HLA-DR antibody (**Figure 2**).

## Discussion

To our knowledge, this prospective study is the first to assess pregnancy outcomes in women with infertility receiving ART in relation to anti-β2GPI/HLA-DR antibody status and explore whether antithrombotic therapy influences pregnancy outcomes in the antibody-positive group. The prevalence of RIF was higher, whereas the implantation rate per ET was significantly lower in the antibody-positive group than in the antibody-negative group. However, in the SET-only analyses, this difference attenuated to a non-significant trend, and the clinical pregnancy rate also tended to be lower than in the antibody-negative group. Clinical pregnancy and live birth rates increased in antibody positive women when treated with LDA/UFH. These data indicate an association between anti-β2GPI/HLA-DR antibodies and severe infertility and suggest the need for more investigations to determine whether LDA/UFH therapy may help restore fecundity in the antibody-positive group.The immunohistochemical study of the eutopic endometrium in the antibody-positive group showed the coordinated expression of β2GPI and HLA-DR antigens in the endometrium. The β2GPI/HLA-DR antigen expression in decidual endothelial cells obtained from women with obstetric APS, which was identified by *in situ* proximity ligation assay, suggested the involvement of anti-β2GPI/HLA-DR antibodies in thrombogenesis (16). Anti-β2GPI/HLA-DR antigen expression in the uterus with the presence of serum autoantibodies may cause intrauterine inflammation, increase platelet function, and promote thrombus formation in women with infertility.

Aspirin and heparin therapies can reduce inflammation, increase uterine perfusion (17, 18), exhibit antithrombin and anticoagulation activities (19), and may be effective in improving clinical pregnancy rates. The efficacy of aspirin and heparin therapies was investigated in women with infertility and implantation failure; however, they did not improve the clinical pregnancy rates (20–23). Given the complicated pathogenesis, some women with implantation failure are anticipated to benefit from aspirin and heparin therapies, whereas others would not, necessitating the development of a test to identify the population who would benefit from antithrombotic therapies. Aspirin and heparin therapies may be effective, particularly for women with infertility and anti-β2GPI/HLA-DR antibody positivity. Recently, a prospective study demonstrated that LDA/UFH therapy increased the live-birth rates and reduced obstetric complications, such as preterm delivery and preeclampsia, in women with RPL and anti-β2GPI/HLA-DR antibodies (24). Similarly, this treatment may effectively increase the live-birth rates among women with infertility and anti-β2GPI/HLA-DR antibodies.

Conversely, the origin of uterine inflammation that induces β2GPI/HLA-DR antigen expression is still unknown. Bacterial infection is considered one of the most plausible causes of intrauterine inflammation. The advent of 16S rRNA sequencing has promoted research on the endometrial microbiome (25, 26). The prevalence of anaerobic bacteria was higher, and the abundance of *Lactobacillus* species was lower in the uterine endometrium of women with implantation failure (27). Thus, whether inflammation caused by abnormal endometrial microbiome is causally associated with β2GPI/HLA-DR antigen expression should be evaluated in future studies.

Recently, Mori et al. discovered a novel pathological mechanism in systemic lupus erythematosus, in which approximately 10% of aberrantly activated T cells misrecognize neoself antigens as nonself and attack them (28). β2GPI/HLA-DR neoself antigens expressed in the endometrium may be the primary target of autoreactive T cells in women with infertility and anti-β2GPI/HLA-DR antibodies. This mechanism should be elucidated in future studies.

In this study, although no significant difference in clinical pregnancy rates per ET was observed between women with and without the antibodies, the implantation rate per ET was significantly lower in the antibody-positive group, suggesting that the presence of the antibodies may affect the implantation process to some extent. Conversely, among women with RIF, no significant differences were found in implantation rates between the antibody-positive and antibody-negative groups, nor between treated and untreated antibody-positive groups. These findings indicate that RIF is a multifactorial condition influenced by various factors, such as maternal age, embryo quality, and endometrial environment, making it difficult to isolate the effect of the antibody alone. The small sample size of RIF cases may also have limited the statistical power of the analysis. Therefore, this study could not demonstrate a clear association between anti-β2GPI/HLA-DR antibody positivity and RIF outcomes. Thus, further large-scale and well-designed studies incorporating multiple RIF-related factors are warranted to elucidate the role of anti-β2GPI/HLA-DR antibodies in implantation and ART outcomes.

This study has several limitations. First, the samples of women with infertility who tested positive for anti-β2GPI/HLA-DR antibodies and treatment subgroups were small. Despite multivariable analyses adjusting for background factors, the statistical power to evaluate treatment effects remains limited; thus, the estimates should be interpreted with caution. Second, treatment allocation was based on physician discretion rather than randomization; therefore, residual confounding cannot be completely excluded despite adjustment. Third, embryos were selected according to institutional criteria; however, their morphological quality varied, and PGT-A was not routinely performed, which may have affected outcomes. Although some differences did not reach statistical significance, likely due to the limited sample size, the observed numerical trends may still reflect clinically meaningful differences, as discussed by previous studies highlighting the potential discrepancy between statistical and clinical significance in small samples (29). Importantly, the relationships observed in this study support association with anti-β2GPI/HLA-DR antibody positivity and do not establish causality or therapeutic efficacy. Thus, future large-scale, multicenter randomized trials or well-controlled prospective studies with standardized embryo selection and SET protocols are warranted to validate our findings and test causality and elucidate the mechanisms linking anti-β2GPI/HLA-DR antibodies to ART outcomes.

## Conclusions

This prospective study for the first time demonstrated that LDA/UFH therapy for women with such antibodies was associated with higher clinical pregnancy rates following ART.

The efficacy and action mechanisms of aspirin and heparin therapies, particularly in women with infertility who test positive for anti-β2GPI/HLA-DR antibodies, warrant further investigation. Future studies should not only include larger samples across multiple centers but also elucidate the biological pathways by which these antibodies affect implantation.

## Data Availability

The original contributions presented in the study are included in the article/**Supplementary Material**. Further inquiries can be directed to the corresponding author/s.
